# A Novel PDE4D Inhibitor BPN14770 Reverses Scopolamine-Induced Cognitive Deficits via cAMP/SIRT1/Akt/Bcl-2 Pathway

**DOI:** 10.3389/fcell.2020.599389

**Published:** 2020-12-10

**Authors:** Yulu Wang, Shichao Gao, Victor Zheng, Ling Chen, Min Ma, Shichen Shen, Jun Qu, Hanting Zhang, Mark E. Gurney, James M. O’Donnell, Ying Xu

**Affiliations:** ^1^College of Pharmacy, Fujian University of Traditional Chinese Medicine, Fuzhou, China; ^2^Department of Pharmaceutical Sciences, School of Pharmacy and Pharmaceutical Sciences, University at Buffalo, State University of New York, Buffalo, NY, United States; ^3^Hangzhou First People’s Hospital, Zhejiang University School of Medicine, Hangzhou, China; ^4^Department of Behavioral Medicine and Psychiatry, Blanchette Rockefeller Neurosciences Institute, West Virginia University Health Sciences Center, Morgantown, WV, United States; ^5^Department of Physiology and Pharmacology, Blanchette Rockefeller Neurosciences Institute, West Virginia University Health Sciences Center, Morgantown, WV, United States; ^6^Department of Cell Stress and Biophysical Oncology, Roswell Park Comprehensive Cancer Center, Buffalo, NY, United States; ^7^Tetra Therapeutics, Grand Rapids, MI, United States

**Keywords:** PDE4D, BPN14770, memory, humanized PDE4D mice, cAMP/SIRT1/Akt/Bcl-2 pathway

## Abstract

A global, quantitative proteomics/systems-biology analysis of the selective pharmacological inhibition of phosphodiesterase-4D (PDE4D) revealed the differential regulation of pathways associated with neuroplasticity in memory-associated brain regions. Subtype selective inhibitors of PDE4D bind in an allosteric site that differs between mice and humans in a single amino acid (tyrosine vs. phenylalanine, respectively). Therefore to study selective inhibition of PDE4D by BPN14770, a subtype selective allosteric inhibitor of PDE4D, we utilized a line of mice in which the PDE4D gene had been humanized by mutating the critical tyrosine to phenylalanine. Relatively low doses of BPN14770 were effective at reversing scopolamine-induced memory and cognitive deficits in humanized PDE4D mice. Inhibition of PDE4D alters the expression of protein kinase A (PKA), Sirt1, Akt, and Bcl-2/Bax which are components of signaling pathways for regulating endocrine response, stress resistance, neuronal autophagy, and apoptosis. Treatment with a series of antagonists, such as H89, sirtinol, and MK-2206, reversed the effect of BPN14770 as shown by behavioral tests and immunoblot analysis. These findings suggest that inhibition of PDE4D enhances signaling through the cAMP-PKA-SIRT1-Akt -Bcl-2/Bax pathway and thereby may provide therapeutic benefit in neurocognitive disorders.

dqaffilConfirm that all author affiliations are correctly listed. Note that affiliations are listed sequentially as per journal style and requests for non-sequential listing will not be applied.

## Introduction

Alzheimer’s disease (AD) is characterized by progressive decline in learning and memory with accompanying neuropathological changes. The disease is a major cause of disability and mortality in aged people. Currently, epidemiological investigations suggest that approximately 44 million people worldwide live with cognitive deficits associated with AD (Van Cauwenberghe et al., 2016). That number will more than triple by 2050 Although the need is great, there are still no disease-modifying therapeutics for AD.

Clinical investigation indicates the therapeutic potential for inhibition of phosphodiesterase-4 (PDE4) for neuropsychiatric disorders (DaSilva et al., 2002). However, dose-limiting side effects of existing inhibitors, which inhibit all PDE4 subtypes with equal potency, has stalled drug development in this area. More recently, it has been possible to design allosteric inhibitors that display PDE4 subtype selectivity, however, in the case of phosphodiesterase-4D (PDE4D), the allosteric binding site is unique to primates, and thus humans (Burgin et al., 2010). A single amino acid difference in UCR2, a phenylalanine in PDE4D and a tyrosine in PDE4A, B, and C, has allowed the design of PDE4D subtype-selective allosteric inhibitors. Based on the unique binding pose of BPN14770 to primate PDE4D, we humanized the mouse PDE4D gene by knocking into C57bl/6 mouse embryonic stem cells a single-nucleotide substitution that replaces UCR2 tyrosine 271 by phenylalanine. Therefore, a line of humanized PDE4D mice was created by altering a single amino acid in the allosteric site from a tyrosine (found in mice) to a phenylalanine (found in human; Zhang et al., 2018). That allowed us to explore in mice the neurochemical and memory-enhancing effect of BPN14770, a prototypical PDE4D allosteric inhibitor. Mutation of the allosteric site increases the potency and selectivity of BPN14770 in the humanized PDE4D mice as compared to wild-type (WT) littermates.

Phosphodiesterase-4D is highly expressed in the cortex and hippocampus (Johansson et al., 2012), integral parts of the limbic system which are implicated in emotional disorders and cognitive dysfunction (Baldi and Bucherelli, 2015). Our previous studies suggested that inhibition of PDE4D by knockdown of PDE4D expression in the hippocampus produced an antidepressant-like effect via upregulation of a cAMP-dependent neuroprotective pathway (Zhang et al., 2002; Wang Z. Z. et al., 2013). More recent work found that the subtype selective, PDE4D allosteric inhibitor, BPN14770 (Gurney et al., 2019) was able to improve memory and cognitive deficits in a mouse model of AD (Zhang et al., 2018). In this study, we explored the role of a downstream signaling module that includes protein kinase A (PKA), sirtuin 1 (Sirt1), and Akt in mediating the effect of PDE4D inhibition by BPN14770. Earlier studies have shown that pharmacological inhibition of PDE4 by rolipram impacts on the Sirt1 pathway by promoting the phosphorylation of Akt (Li et al., 2018). Phosphorylated Akt is known to inhibit several pro-apoptotic Bcl-2 family members, such as Bax. It also activates survival transcription factor nuclear factor kappa-light-chain-enhancer of activated B cells (NF-κB), which up-regulates the pro-survival gene Bcl-2 (Hein et al., 2014; McIlwain et al., 2015). Based on these observations and the evidence that programmed cell death is attributed to common neurological disorders, it is reasonable to hypothesize that PDE4 plays a vital part in the learning and memory deficits that manifest in patients with AD.

In the present study, quantitative proteomics analysis was used to identify proteins whose expression was altered by the PDE4D inhibitor BPN14770 and the biochemical processes associated with these proteins. This supports the hypothesis that PDE4D modulates the Sirt1-Akt pathway in ways relevant to learning and memory. Scopolamine- impairment reduces Sirt1 and Akt expression in the hippocampus and this is reversed by the selective inhibition of PDE4D by BPN14770. We also show that scopolamine-impairment reduces the ratio of Bcl-2/Bax in hippocampus and that this too is reversed by BPN14770. The cognitive benefit of BPN14770 in the scopolamine-impairment model is blocked by pre-treatment with inhibitors of PKA (H89), Sirt1 (sirtinol), and Akt (MK-2206). This suggests that the PKA-SIRT1-Akt pathway is a potential therapeutic target for BPN14770 when it comes to protecting subjects from memory and cognitive impairment such as occurs in AD.

## Materials and Methods

### Animals

Phosphodiesterase-4D humanized mice (C57BL/6 strain) and littermate (WT) controls were produced by InGenous Targeting Laboratory (Ronkonkoma, NY, United States) under contract to Tetra Discovery Partners, Inc. and bred at the University at Buffalo (Zhang et al., 2018). They were housed under controlled ambient temperature (23 ± 1°C) with appropriate humidity (50 ± 10%) and a standard 12 h light/12 h dark cycle. Behavioral and neurochemical analysis was performed with male and female mice at 2–3 months of age (18–25 *g*). The experimental procedures were carried out following the National Institutes of Health Guide for Care and Use of Laboratory Animals (Publication No. 85-23, revised 1985), and approved by the Institutional Animal Care and Use Committee at University at Buffalo, The State University of New York.

### Drug Preparation and Administration

The selective PDE4D allosteric inhibitor BPN14770 (CAS # 1606974-33-7) was synthesized as described (Gurney et al., 2019). BPN14770 was prepared as a 20 × stock solution in 0.5% dimethyl sulfoxide and water. For administration by oral gavage, the stock solution was diluted into 5% Solutol and 90% distilled water for administration at doses of 0.003, 0.01, and 0.03 mg/kg. The selective PKA inhibitor H89 (Sigma-Aldrich, United States) and the SIRT1 and Akt inhibitors, sirtinol and MK-2206 (Selleck Chemicals, United States), were dissolved in 0.9% sterile saline for administration by intraperitoneal injection at doses of 5, 5, and 100 mg/kg, respectively. Scopolamine and rolipram (Sigma-Aldrich, United States) were administered by intraperitoneal injection at doses of 1 and 0.5 mg/kg, respectively, in 0.9% sterile saline.

### Quantitative Proteomics/Systems-Biology Analysis

For the proteomics analysis, male and female humanized PDE4D mice were dosed once daily for 5 days with BPN14770 or vehicle and the brains were harvested 1 h after the last dose. The mice were sacrificed by cervical dislocation, the hippocampus and frontal cortex were harvested, weighed, and then stored in a cryo-freezer set to –80°C.

**FIGURE 1:**
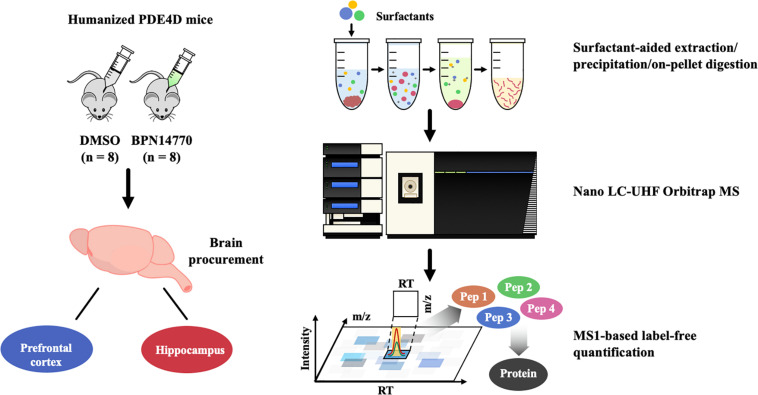
Proteomics analysis flow chart of BPN14770 treatment in humanized PDE4D mice.

The flow chart for proteomics analysis of humanized PDE4D treated with BPN14770 or vehicle is shown in Figure 1. Tissues collected for analysis were the hippocampus and frontal cortex. Brain samples were processed using surfactant-aided precipitation and on-pellet digestion with trypsin (SEPOD). This has been shown previously to yield high sensitivity and consistency in protein quantification by peptide identification (An et al., 2015). After digestion, the samples were analyzed by a high-resolution, high-sensitivity nano LC-Ultra-high-field (UHF) Orbitrap MS system with optimized settings, and protein quantification was conducted with the IonStar data processing pipeline to achieve reproducible MS1-based proteomic quantification (e.g., Supplementary Data Table 1 lists the trypic peptides identified for the PDE4 enzymes). Detailed about the liquid chromatography–mass spectrometry (LC–MS) and quantification methods can be found in previous publications (Nouri-Nigjeh et al., 2014; Shen et al., 2017, 2018). Principal Component Analysis (PCA) and Intra-Group CV were performed on the quantification results to compare the inter-group and intra-group variability of different groups. Data are expressed as the log 2 of the integrated peak height summed across all of the tryptic peptides identified for each protein (Supplementary Data Table 2).

### Bioinformatic Analysis

Difference protein analysis was performed by R script using the Limma R (Linear models for microarray analysis) package with fold change >1.2 or <0.83, and *P* < 0.05 as the cut-off values for differential protein quantification (Supplementary Data Table 2). Comparisons between groups are shown as a Volcano plot and the interaction of the differentially expressed gene (DEG) sets was obtained in a Venn diagram (Supplementary Data Table 3). The STRING database was used to analyze the protein–protein interactions (PPIs) of the differentially expressed proteins, and the hub genes were counted by R script (Supplementary Data Table 4). The gene ontology (GO) and kyoto encyclopedia of genes and genomes (KEGG) enrichment analysis was performed using the Database for Annotation, Visualization, and Integrated Discovery (DAVID) database (Supplementary Data Table 5). Predicted genes of interest were mapped into DAVID to identify the biological processes and the KEGG pathway involved (Supplementary Data Table 6). The KEGG signaling pathway was obtained from the KEGG database. The AD-related genes were obtained from the GeneCards database (Supplementary Data Table 7).

### Behavioral Analysis

For the Morris Water Maze (MWM) and Step-Down Passive Avoidance tests, male and female humanized PDE4D mice were treated by oral gavage with BPN14770 or vehicle once daily for 5 days and their behavior was assessed each day 1 h after dosing. Scopolamine was administered by intraperitoneal injection 30 min before administration of BPN14770 or vehicle. For behavioral assessment in the scopolamine-impairment tests, male and female mice were randomly divided into six groups (10 mice per group) as follows: a control group with no treatment, a scopolamine-impaired group treated with vehicle by oral gavage, groups treated with BPN14770 at 0.003 mg/kg (low dose), 0.01 mg/kg (medium dose), and 0.03 mg/kg (high dose), and a positive control group treated with rolipram, a reference PDE4 inhibitor. Mice were trained in the MWM task 1 h after dosing on days 1, 2, 3, and 4 with the hidden platform test performed on day 5 at 24 h after the last training session. Mice were trained on the Step-down Passive Avoidance task on days 3 and 4 and then tested for step down avoidance on day 5. The MWM test was performed immediately after the Step- down Passive Avoidance test on days 3, 4, and 5. Brain tissues for immunoblot analysis were harvested immediately after the MWM hidden platform test on day 5. In addition, three groups of mice were pretreated 30 min before administration of the high dose of BPN14770 or vehicle with the PKA inhibitor H89, the SIRT1 inhibitor sirtinol, or the Akt phosphorylation (pAkt) inhibitor MK-2206 once daily for 5 days. Behavioral testing was conducted as above and the brains were harvested immediately after the MWM test on day 5 and processed as above.

#### Step-Down Passive Avoidance Test

The protocol for the Step-Down Passive Avoidance Test followed a recent study with slight modifications (Jafari-Sabet et al., 2018). An apparatus containing five 12.5 cm × 12.5 cm × 20 cm chambers was connected to a current-inducing device. Each chamber contained a 5 cm platform that each mouse was placed on at the start of the test. During the acquisition trials on day 3 and 4, each mouse was given 2 min, during which a 0.8 mA current was electrified the floor which was sufficient to provide a mildly aversive stimuli if the mouse left the platform. The mice received twice training sessions each day, once in the morning and once in the afternoon. Mice were tested for step down avoidance 24 h after the last training session on day 5. The retention trial lasted 2 min, but with no current. The latency to step down, the time the mouse waited before jumping off the platform, was recorded.

#### Morris Water Maze Test

The procedure for carrying out the MWM test was nearly identical to those followed by previous experiments (Bromley-Brits et al., 2011). A circular enclosure measuring 125 cm in diameter contained a platform in one of the quadrants and was filled with water (22°C) until the water level resides approximately 0.5 cm above the platform. The test was divided into two components: Hidden Platform Test and Probe Trials. The first component took place four times daily over a 4-day period during which mice explored the pool until they found the hidden platform that was submerged beneath the water. If the mouse failed to find the platform after 60 s, it was placed onto the platform for 20 s. Acquisition of the task is measured by the latency to find the Hiddent Platform. Memory retention was tested 24 h after the last Hidden Platform trial. The hidden platform was removed for the Probe Trial. In the Probe Trial, the percentage of time in the target quadrant, number of crossings across that quadrant, latency to the platform zone, and average swim speed were recorded.

### Immunoblot Assay

All tissue samples were homogenized in radio-immunoprecipitation assay (RIPA) lysis buffer containing protease and phosphatase inhibitors and centrifuged at 12,000 rpm for 30 min at 4°C. The supernatant was mixed with 2× loading buffer and heated to 95–100°C for 5 min to denature the proteins. Protein concentrations were determined using the bicinchoninic acid (BCA) protein assay. Samples (50 μg protein each) were separated using sodium dodecyl sulphate polyacrylamide gel electrophoresis (SDS-PAGE) before transferring to polyvinylidene fluoride (PVDF) membranes (0.20 μm; Millipore, Billerica, MA, United States). The membranes were incubated with 5% bovine serum albumin, a blocking buffer to eliminate effects of non-specific bindings for 1 h at room temperature. Afterward, the blots were incubated with the primary antibodies overnight at 4°C on a shaker: mouse anti-SIRT1 (1:1000; Abcam), mouse anti-actin (1:1000; Abcam) rabbit anti-Akt (1:10000; Abcam), rabbit anti-phospho-Akt (1:1000; Abcam), rabbit Bcl-2 (1:1000; Abcam), and rabbit anti-Bax (1:1000; Abcam). After being washed with tris-buffered saline and tween 20 (TBST), the membranes were incubated with the secondary antibodies on a shaker: goat anti-mouse IgG-HRP (1:3000) goat anti-rabbit IgG-HRP (1: 3,000; Biosharp) for 60 min at room temperature. Band signal was visualized by the enhanced chemiluminescent (ECL) kit (Millipore) and detected by a fluorescence scanner. The resulting images of the bands in question were visualized by an Image-Lab software device.

### Data Analysis

After the images of the blots were collected, their relative protein amounts were quantified by Image J (NIH shareware). The data from the behavioral tests and Immunoblots were analyzed using GraphPad Prism 7.0. Results are expressed as mean ± standard error of the mean (S.E.M.). All data were analyzed statistically by one-way analysis of variance (ANOVA), followed by Dunnett’s *t*-test. All test results that yielded a *P*-value less than 0.05 were considered statistically significant.

## Results

### The Impact of BPN14770 on the Proteome of the Mouse Brain

**FIGURE 2:**
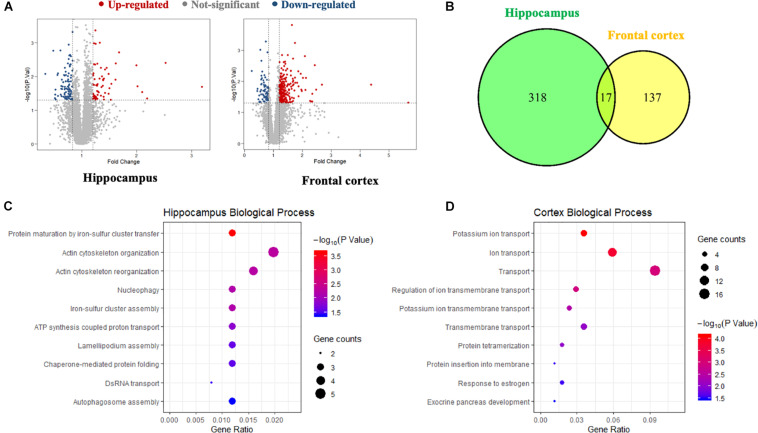
The proteomics analysis of differentially expressed proteins in the hippocampus and frontal cortex samples. **(A)** Volcano plot of differentially expressed proteins in the hippocampus and frontal cortex after BPN14770 treatment (*n* = 8). Fold change >1.2 or <0.83, and *P* < 0.05 as the cut off values for differential protein quantification. **(B)** Venn diagram of hippocampus and frontal cortex differentially expressed proteins. **(C)** The biological processes of gene ontology (GO) enrichment analysis of the differentially expressed proteins in the hippocampus. **(D)** The biological processes of GO enrichment analysis of the differentially expressed proteins in the cortex.

A global, quantitative proteomics/systems-biology analysis showed that inhibition of PDE4D differentially regulated cellular pathways in brain regions associated with memory such as the hippocampus and frontal cortex as shown in the flow chart for the study (Figure 1). Comparison was made between the proteomes of humanized PDE4D mice treated with vehicle or BPN14770 daily for 5 days (0.1 mg/kg, p.o.) using a Volcano plot (Figure 2A). The cuttoff values in the study set the fold change as >1.2 or <0.83 with *P* < 0.05 as the cut off value for statistical signficance. For example, multiple tryptic peptides from PDE4A, PDE4B, and PDE4D were identified and their levels were unchanged in cortex or hippocampus by treatment with BPN14770 (Supplementary Data Tables 1, 2). The number of proteins whose expression differed numbered 325 in the hippocampus (266 up-regulated and 59 down-regulated) and 144 in the frontal cortex (62 up-regulated and 82 down-regulated), Figure 2B. Of these, 17 were in common between the hippocampus and frontal cortex. Based on the GO analysis of biological process, inhibition of PDE4D primarily affected actin cytoskeleton organization and reorganization in the hippocampus (Figure 2C), while in the frontal cortex inhibition of PDE4D affected a series of ion transport systems (Figure 2D).

**FIGURE 3:**
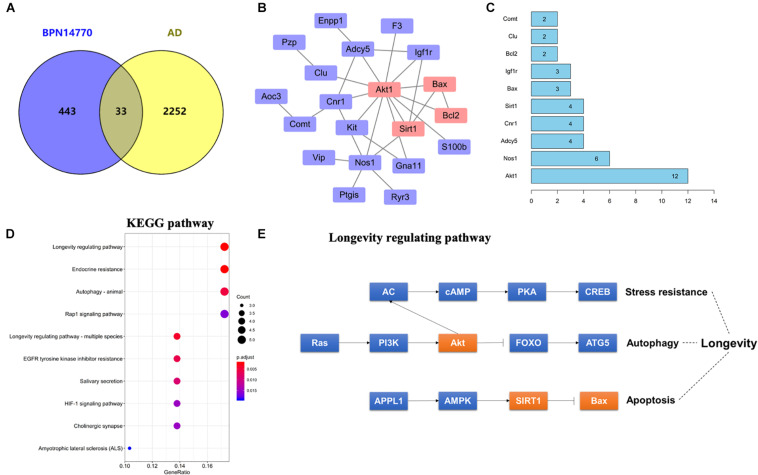
Bioinformatic analysis of differentially expressed proteins and AD-related genes. **(A)** Venn diagram of differentially expressed proteins and AD-related genes. **(B)** The protein–protein interaction (PPI) network of the intersection genes. The red coloring highlights nodal genes that were selected for further analysis by immunoblot. **(C)** Bar plot of the number of hub gene links for each of the nodes in the PPI network. **(D)** KEGG enrichment analysis of intersection genes from the Venn diagram analysis. **(E)** Genes with the KEGG longevity regulating pathway and their potential interaction. Arrows represent an activating effect, T-arrows represent an inhibitory effect. The red nodes are the intersection genes that are regulated by BPN14770 and altered by AD.

In order to explore the relationship between the proteins whose expression was affected by PDE4D inhibition and proteins in the human brain whose expression is affected by AD, a Venn diagram analysis was performed with 2285 AD-related genes obtained from the Genecards database. This showed that 33 of the proteins whose expression was altered by inhibition of PDE4D were also altered in the human brain by AD (Figure 3A). Potential PPI among that set of 33 overlapping proteins were analyzed using the STRING database. The resulting PPI network is shown in Figure 3B. The PPI network analysis identified Akt1 as a hub protein in a network that included Sirt1, Bax, and Bcl2 (Figures 3B,C). Akt1, Sirt1, Bax, and Bcl2 were selected for further analysis due to their relevance to learning and memory disorders. KEGG pathway analysis further suggested that the PPI network proteins were mostly involved in the longevity regulating pathway, endocrine resistance, and autophagy pathways. The top ten hits based on adjusted *P*-value are shown in Figure 3D. As highlighted in Figure 3E, the Sirt1, Akt, and Bax genes comprise a signaling module in the KEGG longevity regulating pathway. This pathway is closely related to neuronal survival and apoptotic processes. The proteomic analysis led to the hypothesis that BPN14770 may impact the Sirt1-Akt-Bax signaling module in neuronal apoptosis and thereby provide potential therapeutic benefit in AD.

### The Effects of BPN14770 on Escape-Learning in the Step-Down Passive Avoidance Test

**FIGURE 4:**
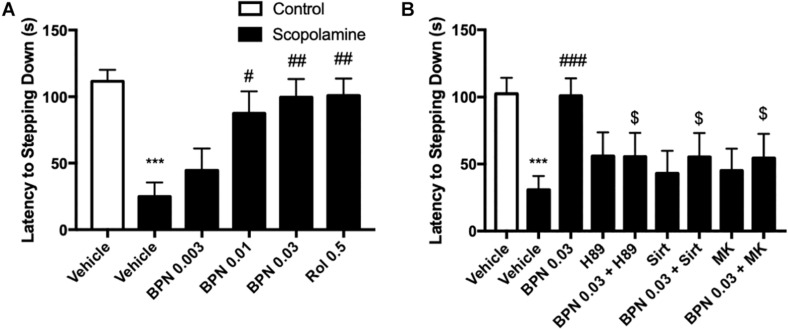
The effects of BPN14770 on aversive memory of Scopolamine treated mice in the step-down passive avoidance test. **(A)** 0.03 mg/kg BPN14770 reversed Scopolamine induced aversive memory impairment. **(B)** H89, sirtinol, or MK-2206 each prevented the effects of BPN14770. Results are displayed as mean ± S.E.M. (*n* = 10 per group). ^∗∗∗^*P* < 0.001 vs. vehicle-treated control group; ^#^*P* < 0.05, ^##^*P* < 0.01, and ^###^*P* < 0.001 vs. vehicle-treated scopolamine group. ^$^*P* < 0.05 vs. BPN-treated scopolamine group. BPN, BPN14770; Rol, rolipram; Sirt, Sirtinol; and MK, MK-2206.

In order to evaluate the effects of BPN14770 in preclinical models of learning and memory disorders, cognitive deficits were induced by treatment with scopolamine 1 h prior to dosing with BPN14770 once daily for 5 days. Mice were evaluated for scopolamine-induced cognitive deficits in the Step-Down Passive Avoidance Model and the MWM. Mice were trained in the Step-Down Passive Avoidance Model on days 3 and 4 of dosing and tested for memory retention on Day 5. The Step-Down Passive Avoidance test was administered immediately prior to the MWM. As shown in Figure 4 for the Step-Down Passive Avoidance test, the average latency to leave the platform was noticeably shorter in mice treated with scopolamine as compared to the vehicle-treated control group (*P* < 0.001). The scopolamine-deficit was reversed in the groups treated with BPN14770 at doses of 0.003, 0.01, and 0.03 mg/kg which exhibited a greater tendency to avoid stepping onto the electrified floor of the chamber as compared to the vehicle-treated scopolamine group [*F*_(4,45)_ = 5.984, *P* = 0.0006], Figure 4A. The Minimum Effective Dose was 0.01 mg/kg p.o. (*P* < 0.05).

The preceding proteomics analysis suggested that the benefit of the PDE4D allosteric inhibitor might be through modulation of cAMP-PKA-CREB and Sirt1-Akt1-Bax signaling modules. Therefore, we explored the effect of inhibiting PKA, Sirt1, or Akt1 on the pharmacological response to BPN14770. H89, a PKA inhibitor, administered 30 min prior to BPN14770 each day blocked the benefit of BPN14770 (*P* < 0.05), further corroborating the idea that PKA-related signaling interacts with

PDE4D inhibitors such as BPN14770 (Figure 1B). Moreover, the SIRT1 inhibitor sirtinol and the Akt phosphorylation inhibitor MK-2206 also blocked the benefit of BPN14770 (*P* < 0.05), Figure 4B.

### The Effects of BPN14770 on Spatial Memory in the Morris Water Maze Test

**FIGURE 5:**
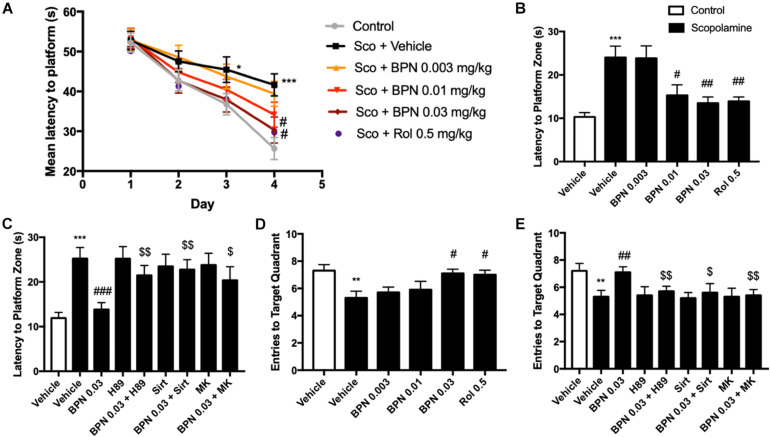
The effects of BPN14770 on spatial learning and memory of Scopolamine treated mice in the Morris Water Maze test. **(A)** Learning curve in the acquisition trials of the Morris Water Maze test from sub-acute treatment of different doses of BPN14770. BPN14770 reversed scopolamine-induced spatial memory deficit 24 h after the acquisition trials. In the probe trials, the latency to platform location **(B,C)**, entries into the target quadrant **(D,E)** after treatment with BPN14770 and different inhibitors. H89, Sirtinol, and MK-2206 each prevented the effects of 0.03 mg/kg BPN14770. Results are displayed as mean ± S.E.M. (*n* = 10 per group); ^∗^*P* < 0.05, ^∗∗^*P* < 0.01, and ^∗∗∗^*P* < 0.001 vs. vehicle-treated control group; ^#^*P* < 0.05, ^##^*P* < 0.01, and ^###^*P* < 0.001 vs. vehicle-treated scopolamine group; ^$^*P* < 0.05 and ^$$^*P* < 0.01 vs. BPN-treated scopolamine group. Rol, rolipram; Sco, Scopolamine; Sirt, Sirtinol; and MK, MK-2206.

The MWM test was administered 1 hr after dosing with BPN14770 or vehicle over five successive days. On day 1, all groups took similar time to find the platform. However, by days 3 and 4 the scopolamine-treated mice took considerably longer to reach the platform on as compared to their vehicle-treated littermates (*P* < 0.05; *P* < 0.001; Figure 5A). This deficit was reduced by daily treatment with BPN14770. By day 4, treatment with BPN14770 dose-dependently reduced the average time required for the scopolamine-impaired mice to reach the hidden platform [*F*_(4,45)_ = 5.955, *P* = 0.0006] at doses of 0.01 mg/kg (*P* < 0.05) and 0.03 mg/kg p.o. (*P* < 0.01), Figure 5B. The benefit of BPN14770 in scopolamine-impaired mice was prevented by the PKA inhibitor H89, the SIRT1 inhibitor sirtinol, and the pAkt inhibitor MK-2206 (*P*’s < 0.01; or *P* < 0.05, Figure 5C). No treatment altered swimming velocity (data not shown).

Scopolamine also causes a deficit in retention of the location of the hidden platform as shown in the Probe Trial on day 5. The number of entries into the target quadrant as the mice searched for the missing platform was reduced for the scopolamine-impaired group treated with vehicle (*P* < 0.01, Figure 5D). This deficit was reversed by BPN14770 [F _(4,45)_ = 3.25, *P* = 0.02] at a dose of 0.03 mg/kg p.o. (*P* < 0.05). The inhibitors H89, sirtinol, and MK-2206 counteracted this benefit of BPN14770 (*P* < 0.05 or *P* < 0.01, Figure 5E).

### The Effects of BPN14770 on SIRT1 Expression in the Hippocampus

**FIGURE 6:**
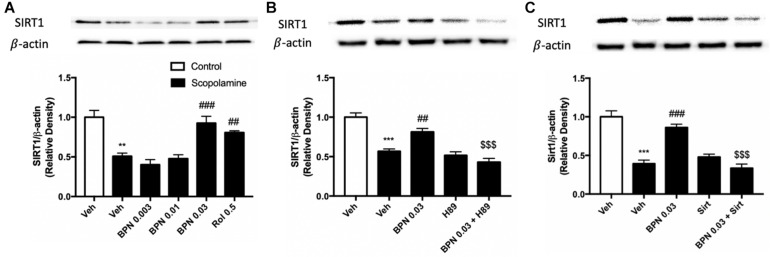
Immunoblot analysis for SIRT1 expression in the hippocampus. SIRT1 expression was downregulated after scopolamine treatment, which was reversed by 0.03 mg/kg BPN14770 **(A)**. Pre-treatment with H89 **(B)** or Sirtinol **(C)** prevented the therapeutic effects of BPN14770. Results are displayed as mean ± S.E.M. (*n* = 4–5 per group). ^∗∗^*P* < 0.01 and ^∗∗∗^*P* < 0.001 vs. vehicle-treated control group; ^##^*P* < 0.01 and ^###^*P* < 0.001 vs. vehicle-treated scopolamine group; ^$$$^*P* < 0.001 vs. BPN-treated scopolamine group. Rol, rolipram; Sirt, Sirtinol.

To confirm and extend the proteomic and behavioral analyses, we used immunoblot analysis to probe the regulation of Sirt1 expression in the hippocampus. This would allow us to clarify the possible mechanism of BPN14770 in relation to the amelioration of the cognitive deficits induced by scopolamine. The experiment revealed that Sirt1 expression is decreased in the hippocampus after exposure to scopolamine, as shown in Figure 6A (*P* < 0.01). However, the decrease in SIRT1 expression in the hippocampal was ameliorated by treatment with BPN14770 at 0.03 mg/kg [*F*_(4,15)_ = 16.94, *P* = 0.0001]. The effects of BPN14770 on SIRT1 expression were reversed by pre-treatment with H89 or sirtinol (*P* < 0.001; Figures 6B,C). Treating mice exposed to scopolamine with only an inhibitor (H89 or Sirtinol) without BPN14770 did not change Sirt1 expression in comparison to the group treated with scopolamine exclusively.

### The Effects of BPN14770 on the Ratio of Phosphorylated Akt to Total Akt in the Hippocampus

**FIGURE 7:**
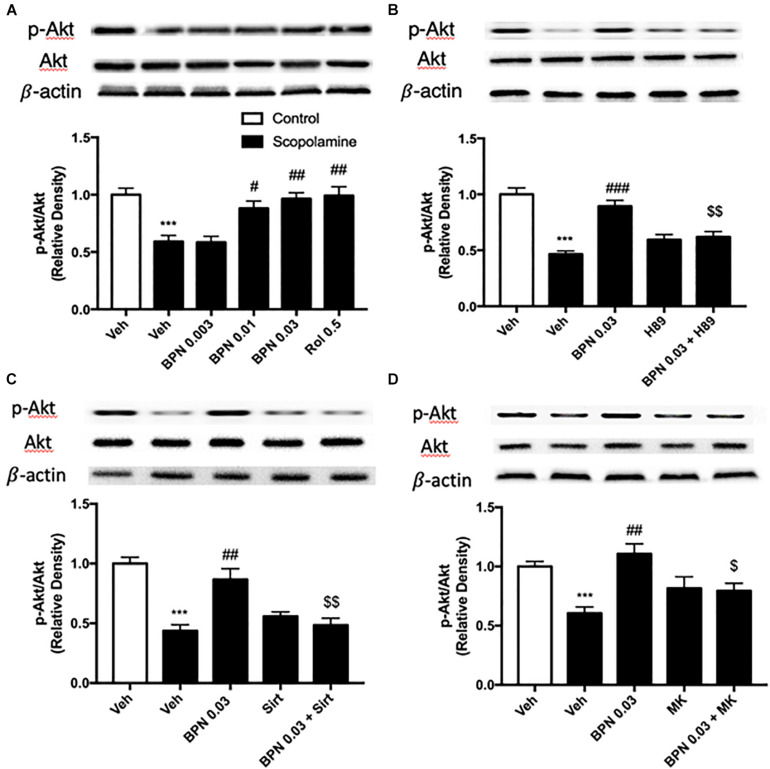
Immunoblot analysis for the ratio of p-Akt to Akt in the hippocampus. The ratio of p-Akt to Akt is decreased after scopolamine treatment, but reversed by 0.01 and 0.03 mg/kg BPN14770 **(A)**. Pre-treatment with H89 **(B)**, Sirtinol **(C)**, or MK-2206 **(D)** prevented the therapeutic effects of BPN14770. Results are displayed as mean ± S.E.M. (*n* = 4–5 per group). ^∗∗∗^*P* < 0.001 vs. vehicle-treated control group; ^#^*P* < 0.05, ^##^*P* < 0.01, and ^###^*P* < 0.001 vs. vehicle-treated scopolamine group; ^$^*P* < 0.05 and ^$$^*P* < 0.01 vs. BPN-treated scopolamine group. Rol, rolipram; Sirt, Sirtinol; and MK, MK-2206.

As shown in Figure 7A, exposure to scopolamine did not affect total Akt levels but significantly decreased the levels of phospho-Akt (pAkt) in the hippocampus as compared to the vehicle-treated control group (*P* < 0.001). The reduction in the ratio of pAkt to Akt was reversed by administering BPN14770 at 0.01 and 0.03 mg/kg p.o. (*P* < 0.05; *P* < 0.01, respectively). The fact that the ratio was higher for the greater concentrations of the drug implies a dose-dependent therapeutic effect [*F*_(4,15)_ = 10.82, *P* = 0.0002 in the hippocampus]. Furthermore, pretreated either H89, Sirtinol, or MK-2206 prevented the increase in hippocampal p-Akt to Akt ratio bestowed by BPN14770 (*P* < 0.01 or *P* < 0.05; Figures 7B–D).

### The Effects of BPN14770 on Bcl-2 and Bax Expression in the Hippocampus

**FIGURE 8:**
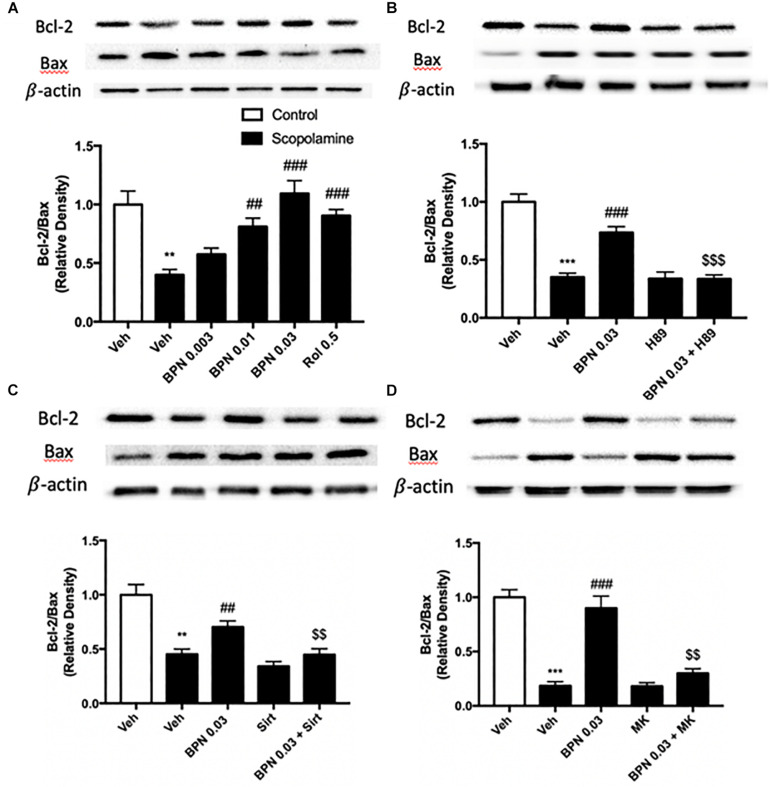
Immunoblot analysis for the ratio of Bcl-2 to Bax in the hippocampus. The ratio of Bcl-2 to Bax is decreased after scopolamine treatment, but reversed by 0.01 and 0.03 mg/kg BPN14770 **(A)**. Pre-treatment with H89 **(B)**, sirtinol **(C)**, or MK-2206 **(D)** prevented the therapeutic effects of BPN14770 on protein expression. Results are displayed as mean ± S.E.M. (*n* = 4–5 per group). ^∗∗^*P* < 0.01 and ^∗∗∗^*P* < 0.001 vs. vehicle-treated control group; ^##^*P* < 0.01 and ^###^*P* < 0.001 vs. vehicle-treated scopolamine group; ^$$^*P* < 0.01 and ^$$$^*P* < 0.001 vs. BPN-treated scopolamine group. Rol, rolipram; Sirt, sirtinol; and MK, MK-2206.

The B-cell lymphoma 2 (Bcl-2) to Bax ratio is an indicator of the extent of apoptosis in the target tissue. In our study, the proteomics results showed that Bax was up-regulated in the hippocampus, and Bcl2 was down-regulated in the cortex after treatment with BPN14770, which did not meet our prediction (Supplementary Data Table 2). To verify the proteomics results, the Bax and Bcl2 protein expression were detected in the hippocampus after treatment with BPN14770. We found that Bax was down-regulated, and Bcl2 was up-regulated in the hippocampus, which were inconsistent with proteomics results. In addition, by analyzing the KEGG pathways of differential

genes, we found that the apoptosis-related pathway is enriched in scopolamine treated mice in the presence of BPN14770, which provide further evidence for our hypothesis. In the present study, a substantial reduction in the Bcl-2/Bax ratio was observed in the hippocampi of mice treated with scopolamine (*P* < 0.01) as shown in Figure 8A. Administering BPN14770 increased the Bcl-2/Bax ratio dose-dependently in the hippocampus [*F*_(4,15)_ = 14.83, *P* < 0.0001]. At 0.01 or 0.03 mg/kg doses, the average ratio of Bl-2/Bax was close to that of the vehicle-treated control mice (*P* < 0.01; *P* < 0.001, respectively). The PKA inhibitor H89 reversed the BPN14770-induced increase in Bcl-2/Bax ratio (*P* < 0.001, Figure 8B) as did sirtinol (Figure 8C), and MK-2206 (Figure 8D), *P* < 0.01.

## Discussion

Nausea and emesis are the major side effects that limit the tolerability of PDE4 inhibitors in a therapeutic setting. Our previous study (Zhang et al., 2018) examined the duration of ketamine/xylazine-induced anesthesia as a pharmacological surrogate for assessment of emetic potential as commonly seen in other species. PDE4B and PDE4D gene-deleted mice differ by this measure, with PDE4D gene-deleted being more prone to “emesis” by this surrogate. Comparison of WT and humanized PDE4D mice demonstrated that BPN14770 dose dependently reduced the duration of ketamine/xylazine anesthesia. In our pilot separate experiments, plasma exposure at the NOAEL (no observed adverse effect level) of 1 mg/kg PO with BPN14770 was 1305 ± 113 ng/ml at 1 h post dose. Cognitive benefit and elevation of PKA-CREB pathway related biomarkers were shown to occur at plasma exposures of 10–30 ng/ml. Thus, there is a 40- to 100-fold window in exposure between doses of BPN14770 in the humanized PDE4D mice that benefit neurochemical and cognitive biomarkers of PKA-CREB pathway outflow as compared to the plasma expos.

**FIGURE 9:**
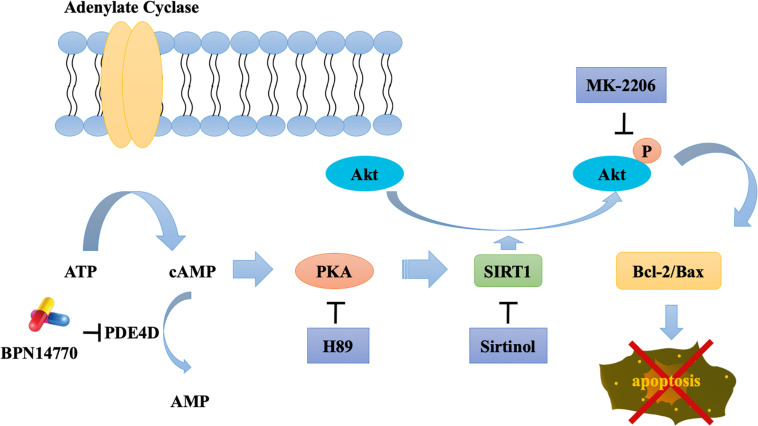
Schematic outline of the probable mechanisms behind BPN14770-induced anti-apoptosis of neurons.

PDE4A, PDE4B, and PDE4D are expressed in the brain regions such as the prefrontal cortex, hippocampus, amygdala, and nucleus accumbens (Iona et al., 1998; Cherry and Davis, 1999). They serve as different roles in the central nervous system because of their distribution pattern and subcellular compartmentalization of each subtype, which provide a theoretical basis for the separation of therapeutic and adverse effects of PDE4 inhibitors. PDE4A or PDE4B deficient mice display antidepressant-like behavior, while PDE4D knockout mice exhibit improved learning and memory properties (Zhang et al., 2002). Recently, the discovery of subtype selective, allosteric PDE4D inhibitor BPN14770 has achieved much progress with the use of x-ray crystallography (Zhang et al., 2017), which suggest that BPN14770 binds in the catalytic site of PDE4 that allows closure of upstream conserved regions 2 (UCR2). Closure of UCR2 inhibits the access of cAMP to the catalytic site and consequently enzymatic activity, resulting in memory enhancing effects. In the present study, the proteomics analysis with label-free protein quantification suggested that hundreds of proteins were impacted by PDE4D inhibition by BPN14770 at a dose consistent with the mitigation of scopolamine-induced deficits in learning and memory. Our studies with inhibitors of key signaling components indicates the benefit of BPN14770 is mediated through modulation of cAMP-PKA-SIRT1-Akt-Bcl-2/Bax signaling (Figure 9). These findings suggest that BPN14770 may have benefit in the treatment of memory deficits in neurodegenerative disorders such as AD.

A global, quantitative proteomics/systems-biology analysis indicated that 325 proteins in the hippocampus and 144 proteins in the frontal cortex were significantly altered in expression after BPN14770 treatment. The gene ontology enrichment analysis indicated that PDE4D inhibition modulates actin cytoskeleton organization and reorganization, and nucleophagy in the hippocampus, while a serial of ion transport items were affected in the frontal cortex. Further bioinformatic analysis revealed that 33 of the proteins whose expression was altered by BPN14770 were closely altered in AD. PPI analysis indicated a potential connection among the Sirt1, Akt, Bax, and Bcl-2 genes, all of which are involved in governing neuronal cell death. This leads to the theory that a signaling cascade involving the proteins extrapolated from the gene map is responsible for regulation of cAMP-PKA-SIRT1-Akt -Bcl-2/Bax pathway.

Scopolamine-induced memory deficit is a classical animal model of the cholinergic deficit in AD due to the selective death of cholinergic neurons in the basal forebrain (Kim et al., 2013). The behavioral tests suggested that administration of scopolamine simulates symptoms associated with loss of synaptic plasticity, such as learning and memory deficits. Previous studies in scopolamine-impairment model indicated that acute treatment with the PDE4D inhibitor BPN14770 resulted in improvement in short-term spatial memory in a dose-dependent manner in the Y-maze test (Zhang et al., 2018). In the present study, the outcomes of the step-down passive avoidance test suggested that sub-acute treatment with BPN14770 for 5 days ameliorated scopolamine-induced loss of aversive memory. These beneficial effects of BPN14770 on scopolamine-treated mice were prevented by the administration of either a PKA inhibitor (H89), a SIRT1 inhibitor (sirtinol), or a pAkt inhibitor (MK-2206), which supports the hypothesis that cAMP, PKA, Sirt1, and Akt signaling pathways may be important for the action of the drug. The benefit of BPN14770 was seen after 5 days of dosing but was not seen after a single dose (data not shown).

Immunoblots analysis was performed to explore the possible mechanism behind the benefit of BPN14770 in the models of scopolamine-impaired learning and memory. Enhancing signaling in the cAMP/PKA pathway via PDE4 inhibition can increase the intrinsic catalytic function of Sirt1 through S434 phosphorylation (Gerhart-Hines et al., 2011). Previous studies have shown that rolipram increases cAMP levels and activates Epac1, a cAMP sensor (Park et al., 2012). This leads to activation of the CamKKβ-AMPK pathway which increases intracellular Ca^2+^ levels as well as NAD^+^ and Sirt1 expression. Other studies suggest that sirtuins 1–3 possess are NAD-dependent deacetylases, which deacetylate downstream substrates including tau, lead to proteasomal degradation and subsequently reduce aggregation of tau in the AD brain (Herskovits and Guarente, 2014). Our previous studies indicated that the inhibition of PDE4 by rolipram is responsible for preventing the break down cyclic cAMP into AMP (Zhang et al., 2017). BPN14770 has demonstrated that it affects cAMP levels in the same manner as rolipram, so that it might have an impact on Sirt1 involved neuronal cell preservation in neurodegenerative disorders is plausible. Although the beneficial role of Sirt1 has been evaluated across the context of multiple diseases such as inflammation and insulin resistance (Yoshizaki et al., 2009), the process through which it prevents apoptosis requires further investigation.

Akt, also known as Protein Kinase B, is a serine-threonine kinase which plays a role in cellular survival mechanisms via inhibiting apoptosis. It is activated by second messenger phospholipid kinase phosphatidylinositol 3-kinase (PI3K) in the PI3K/AKT/mTOR pathway (Zhang et al., 2011). Mammals possess three distinct isoforms of Akt (Akt1, Akt2, and Akt3) that constitute a pleckstrin homology domain, a hydrophobic motif, and a kinase domain. Akt undergoes phosphorylation at Ser473 in the hydrophobic motif and Thr308 in the kinase domain. The pleckstrin homology domain is there to promote interaction with the plasma membrane which is essential for Akt phosphorylation (Calleja et al., 2009). During this step, Akt also phosphorylates downstream targets, some of which contribute to cell survival (Zhang et al., 2011). Akt activation alleviating learning and memory deficits stemming from Aβ is detailed in a study utilizing a mouse model of AD (Yi et al., 2018). Recent studies have corroborated the idea that there is an interaction between Sirt1 and Akt expression. To illustrate, histone acetyltransferases (HATs) transfer an acetyl moiety to Akt on lysine amino acids, inhibiting its phosphorylation. Sirt1 is a histone deacetylase (HDAC) that utilizes NAD to deacetylate Akt on the lysine residues targeted by HATs (Pillai et al., 2014). Given that both expression of Sirt1 and Akt govern neuronal survival (Wang S. et al., 2013) and that PDE4 inhibitors could increase Sirt1 expression, the results of immunoblot showed that SIRT1 and Akt antagonists reversed the effects of BPN 14770, which suggest that BPN 14770 exhibits neuroprotective effects by cAMP-PKA-SIRT1-Akt -Bcl-2/Bax pathway. Our data indicated that BPN14770 treatment greatly increased Sirt1 expression and also led to increased phosphorylation of Akt. Furthermore, administration of sirtinol reversed effects the of BPN14770 on SIRT1 and pAkt expression. It is well known that Bcl-2 is a member of the Bcl-2 family located on the outskirts of the mitochondria that inhibits pro-apoptotic proteins (Khanzadeh et al., 2018). Bcl-2-associated X protein (Bax) is a pro-apoptotic member of the Bcl-2 family that forms an oligomeric pore upon activation, enabling pro-apoptotic factors such as cytochrome c to migrate from the mitochondria to the cytoplasm (Westphal et al., 2014). Thus, the Bcl-2 to Bax ratio is a strong indicator of the extent to which apoptosis may be suppressed with a higher ratio of Bcl-2 to Bax indicating higher protection. Among 476 changed proteins, Bax was up-regulated in the hippocampus, while Bcl2 did not change in the proteomics analysis, which did not meet our prediction. To verify the proteomics results, we used the western blot test to detect the ratio of Bcl2 to Bax in the hippocampus. We found that Bax is down-regulated, while Bcl2 is up-regulated after treatment with BPN14770. The reason is that proteomics is a high-throughput analysis method, the results of which may differ from the actual protein changes due to the sensitivity of this methods to both the preparation processes and sample biology. Thus, it seemed like necessary for the further immunoblot assay to verify the differentially expressed protein in the proteomics assay. In the present study, the Bcl-2/Bax ratio was decreased after administration of scopolamine but increased as BPN14770 dose was raised. However, the three inhibitors, H89, sirtinol, and MK2206, reversed BPN14770’s effects on the ratio of Bcl-2 to Bax, indicating that the antiapoptotic effects of BPN14770 are through PKA dependent SIRT-Akt pathway.

Some studies have shown that cells may have an advanced mechanism that allows mTORC1 to immediately respond to the cAMP signal via the dual function of PDE4D (Kim et al., 2010). The possible negative regulation of PDE4D by Rheb binding under basal conditions and of the cAMP-induced activation of PDE4D may be interesting for future studies aimed at elucidating additional details of the cross talk between the cAMP and mTOR pathways. The interesting finding will allow us to further study the mechanism of BPN14770 on cognition and memory performance in aging.

In conclusion, the results of our study demonstrate that subacute treatment with PDE4D inhibitor BPN14770 is effective at alleviating scopolamine-induced memory and cognitive impairment. The underlying mechanism may be involved in the neuroprotective and antiapoptoticeffects of BPN14770 by regulation of the cAMP-PKA-SIRT1-Akt -Bcl-2/Bax pathway.

## Data Availability Statement

The original contributions presented in the study are included in the article/Supplementary Material, further inquiries can be directed to the corresponding author/s.

## Ethics Statement

The experimental procedures were carried out following the National Institutes of Health Guide for Care and Use of Laboratory Animals (Publication No. 85-23, revised 1985), and approved by the Institutional Animal Care and Use Committee at University at Buffalo, The State University of New York.

## Author Contributions

YW and VZ performed the experiments. SG, LC, and HZ conducted the bioinformatic analyses and wrote the manuscript involved this part. MM, SS, and JQ were responsible for the quantitative proteomics/systems-biology analysis. MG reviewed and edited the manuscript. YX and JO’D conceived and designed the study. All authors contributed to the article and approved the submitted version.

## Conflict of Interest

MG is an employee of Tetra Therapeutics which has a financial interest in BPN14770. The remaining authors declare that the research was conducted in the absence of any commercial or financial relationships that could be construed as a potential conflict of interest.
